# Exploring the Clinical Diversity of Castleman Disease and TAFRO Syndrome: A Japanese Multicenter Study on Lymph Node Distribution Patterns

**DOI:** 10.1002/ajh.27612

**Published:** 2025-01-25

**Authors:** Mizuna Otsuka, Tomohiro Koga, Remi Sumiyoshi, Shoichi Fukui, Yuko Kaneko, Takayuki Shimizu, Atsushi Katsube, Shingo Yano, Yasufumi Masaki, Makoto Ide, Hajime Yoshifuji, Masayasu Kitano, Yasuharu Sato, Naoki Sawa, Hiroaki Niiro, Naoya Nakamura, David C. Fajgenbaum, Frits van Rhee, Atsushi Kawakami

**Affiliations:** ^1^ Department of Immunology and Rheumatology, Division of Advanced Preventive Medical Sciences Nagasaki University Graduate School of Biomedical Sciences Nagasaki Japan; ^2^ Clinical Research Center Nagasaki University Hospital Nagasaki Japan; ^3^ Division of Rheumatology, Department of Internal Medicine Keio University School of Medicine Tokyo Japan; ^4^ Division of Hematology, Department of Medicine Keio University School of Medicine Tokyo Japan; ^5^ Division of Hematology, Department of Medicine National Hospital Organization Tokyo Medical Center Tokyo Japan; ^6^ Division of Clinical Oncology and Hematology, Department of Internal Medicine The Jikei University School of Medicine Tokyo Japan; ^7^ Department of Hematology and Immunology Kanazawa Medical University Kanazawa Japan; ^8^ Department of Hematology Takamatsu Red Cross Hospital Takamatsu Japan; ^9^ Department of Rheumatology and Clinical Immunology, Graduate School of Medicine Kyoto University Kyoto Japan; ^10^ Department of Collagen Disease and Rheumatology Sumitomo Hospital Osaka Japan; ^11^ Department of Molecular Hematopathology Okayama University Graduate School of Health Sciences Okayama Japan; ^12^ Nephrology Center and Department of Rheumatology Toranomon Hospital Kajigaya Tokyo Japan; ^13^ Department of Clinical Immunology and Rheumatology/Infectious Disease Kyushu University Hospital Fukuoka Japan; ^14^ Department of Pathology Tokai University School of Medicine Isehara Japan; ^15^ Center for Cytokine Storm Treatment & Laboratory University of Pennsylvania Philadelphia Pennsylvania USA; ^16^ Myeloma Center University of Arkansas for Medical Sciences Little Rock Arkansas USA

**Keywords:** Castleman disease, clinical variability, lymph node distribution, multicenter cohort, TAFRO syndrome

## Abstract

Individuals diagnosed with Castleman disease (CD) and TAFRO syndrome (characterized by thrombocytopenia, anasarca, fever, bone marrow fibrosis, and organomegaly) displays a wide range of clinical symptoms, including varying patterns of lymph node enlargement, systemic inflammation, and impaired organ function. Some patients may present with both CD and TAFRO syndrome concurrently. A retrospective study conducted across multiple centers in Japan examined 321 cases to determine if the quantity and position of swollen lymph nodes could forecast the clinical progression and intensity of these conditions. Interestingly, the study revealed that patients with TAFRO syndrome exhibited lymphadenopathy across all ranges of lymph node region counts. Moreover, no specific clinical patterns were associated with the number of affected lymph node regions in CD patients, regardless of whether they also had TAFRO syndrome. These results enhance our understanding of the clinical variability in CD and TAFRO syndrome, suggesting that a comprehensive clinical evaluation, rather than relying solely on lymph node count, is crucial for effectively managing these conditions. Additional studies are required to establish reliable diagnostic markers and to predict disease severity at the time of diagnosis, ultimately improving patient outcomes.

## 1 Introduction

Castleman disease (CD) is a lymphoproliferative disorder involving characteristic histopathological features in the lymph nodes [1]. CD is classified as unicentric CD (UCD) or multicentric CD (MCD) depending on the number of enlarged lymph node stations [2]. Histologically, CD can also be divided into the following subtypes: hyaline vascular, which involves atrophic germinal centers, mantle zone expansion, and hyalinized vessels penetrating germinal centers; plasmacytic, which involves sheet‐like proliferation of plasma cells in interfollicular areas with hyperplastic germinal centers; and mixed, which includes features of both. Hypervascular is used to describe when hyaline vascular features (typically found in UCD) are observed in MCD. However, the clinical significance of these pathological classifications is not well understood [3].

MCD is subcategorized according to etiology into human herpesvirus‐8‐associated MCD (HHV‐8‐associated MCD), polyneuropathy, organomegaly, endocrinopathy, monoclonal plasma cell disorder, skin changes (POEMS) syndrome‐associated MCD, and idiopathic MCD (iMCD), where the etiology is unknown. Among iMCD subtypes, cases with thrombocytopenia, anasarca, fever, bone marrow fibrosis, and organomegaly (TAFRO) are classified as iMCD‐TAFRO [4]. Conversely, cases with IgG levels > 3500 mg/dL, elevated platelet counts, and lymph node biopsies demonstrating hyperplastic germinal centers and sheet‐like plasma cell proliferation are classified as the idiopathic plasmacytic lymphadenopathy with hyperimunnogloblinemia subtype (iMCD‐IPL) [5], whereas the remainder of iMCD patients are classified as iMCD not otherwise specified (iMCD‐NOS) [6]. Most of the literature to date on iMCD‐IPL originates from Japan to other Asian countries [6, 7].

iMCD‐TAFRO is considered the most severe subtype of iMCD. It is often associated with normal or slightly elevated gamma globulins [8, 9] and is accompanied by elevated hepatobiliary enzymes, especially the alkaline phosphatase [10]. In terms of pathological findings of lymph nodes, iMCD‐TAFRO is usually characterized by mixed and hypervascular types [8]. iMCD‐IPL frequently exhibit plasmacytic histopathology [11].

Additionally, the existence of TAFRO syndrome without iMCD, which is characterized by TAFRO symptoms with or without small lymphadenopathy, has been reported [4, 12]. The presence of TAFRO symptoms is associated with a poor prognosis, with complications such as unmanageable fluid retention, reduced intravascular volume, and dysfunction of the liver, kidneys, and bone marrow, which ultimately lead to mortality. In TAFRO syndrome, the overall survival has been reported to decline significantly within 24 months of diagnosis, by which one‐third of patients have died [13]. In clinical practice, the presence of TAFRO symptoms should early to the physician to a severe clinical course, the urgent need for early and correct diagnosis as well immediate therapeutic intervention. Due to the poor overall condition of patients and relatively small lymphadenopathy in iMCD‐TAFRO, tissue biopsies for diagnosis can be difficult, resulting in treatment sometimes taking precedence over a confirmed diagnosis. Although histopathology is critical for diagnosis, there is a need for other diagnostic indicators. Moreover, the prediction of disease severity at diagnosis could lead to improved survival rates.

In this context, a 2021 report by Pierson et al. proposed that the number of swollen lymph node regions and their relationship with the diaphragm (i.e., UCD [one swollen region], oligocentric CD [OligoCD; a few regions on one side of the diaphragm], and iMCD [multiple regions spanning both sides of the diaphragm]) may differentiate clinical subtypes and treatment responses [14]. This classification resembles the Ann Arbor staging system for malignant lymphoma, where crossing the diaphragm is a known adverse prognostic factor, suggesting its potential relevance to iMCD [15]. In contrast, in Japan, prognosis is often predicted according to the presence or absence of TAFRO symptoms, IPL, and NOS clinical subtypes [9]. The objectives of this study were to adhere to the Japanese criteria for CD, categorize cases with inflammatory symptoms and findings into four groups according to the number of lymph node regions involved: Group 0 (G0), no swollen lymph nodes; Group 1 (G1), one enlarged lymph node region; Group 2 (G2), swollen lymph nodes on one side of the diaphragm; Group 3 (G3), swollen lymph nodes on both sides of the diaphragm. This investigation aims to determine whether there are differences in severity and clinical characteristics among these groups.

## 2 Materials and Methods

### 2.1 Patient Classification and Definitions

This multicenter, retrospective, cross‐sectional cohort study analyzed data from 332 patients diagnosed with CD or TAFRO syndrome, who had information on enlarged lymph nodes and histopathological findings and were followed up at 11 rheumatology and hematology departments between 1995 and 2022. Participants were recruited from Nagasaki University Hospital, Keio University Hospital, Jikei University Hospital, Kanazawa Medical University Hospital, Takamatsu Red Cross Hospital, Kyoto University Hospital, Sumitomo Hospital, Okayama University Hospital, Toranomon Hospital, and Kyushu University Hospital. This study was registered at the University Hospital Medical Information Network Center (UMIN000046356). The diagnosis of CD was based on the Japanese diagnostic criteria for CD [16] and was made by the Japan College of Rheumatology‐certified rheumatologist or the Japanese Society of Hematology‐certified hematologists who belong to The Intractable Disease Policy Research Project research team or Japan Agency for Medical Research and Development (AMED) Practical Research Project for Rare/Intractable Diseases team for Castleman disease or TAFRO syndrome. Accordingly, we obtained the lymph node histology findings diagnosed by The Japanese Society of Pathology‐certified pathologists (i.e., hyaline vascular type/hypervascular type, plasma cell type, mixed type, respectively) from each rheumatology and hematology department described above and clinical subtypes of CD such as iMCD‐TAFRO, iMCD‐IPL, and iMCD‐NOS were classified by consensus from three Japan College of Rheumatology‐certified rheumatologists (T.K., S.F., R.S. at Nagasaki University belonging to The Intractable Disease Policy Research Project research team and AMED Practical Research Project for Rare/Intractable Diseases team for Castleman disease or TAFRO syndrome) according to the definition described previously (cases with thrombocytopenia, anasarca, fever, bone marrow fibrosis, and organomegaly are classified as iMCD‐TAFRO, cases with serum IgG levels > 3500 mg/dL and lymph node biopsies demonstrating germinal center hyperplasia and sheet‐like plasma cell proliferation are classified as iMCD‐IPL [17], the rest are classified as iMCD‐NOS, respectively) [6, 18]. Inflammatory syndrome was defined as at least two of three conditions: anemia, hypoalbuminemia, and inflammation, defined as hemoglobin < 11.5 g/dL (males) or < 10.5 g/dL (females), albumin < 3.5 g/dL, CRP > 20 mg/L, or ESR > 30 mm/h, respectively. Severity was assessed using the CHAP score. The CHAP score is a composite index derived from C‐reactive protein (CRP), hemoglobin levels, albumin levels, and performance status, with each component scored from 0 to 4 points (in total distributed from 0 to 12 points) to evaluate disease severity [16] (Table S1). TAFRO syndrome was diagnosed using the Japanese diagnostic criteria for TAFRO syndrome [19], and its severity was evaluated according to the TAFRO syndrome severity classification 2019 [19]. The TAFRO syndrome severity classification uses a composite index of fluid retention, platelet count, fever and/or inflammation, and renal impairment, each scoring from 0 to 3 points (in total 0–12 points). The severity was graded as follows: 0–4 points (*mild*, grade 1), 5–6 points (*moderate*, grade 2), 7–8 points (*moderate–severe*, grade 3), 9–10 points (*severe*, grade 4), and 11–12 points (*very severe*, grade 5). This scoring system can also be used to assess disease activity (Table S2).

Based on Pierson et al.'s classification [14] of UCD, OligoCD, and iMCD, we added a type with no lymph node lesions and divided the patients into the following four groups according to the number of lymph node regions: G0, patients with 0 swollen lymph nodes (not CD); G1, CD patients with one enlarged lymph node region (UCD); G2, patients with more than one swollen lymph node region on only one side of the diaphragm (OligoCD); G3, patients with multiple swollen lymph node regions on both sides of the diaphragm (iMCD).

This study analyzed whether there were any associations between the four groups with regard to clinical subtype, lymph node histology, severity, and laboratory findings focusing specifically on data collected at the time of diagnosis, before the initiation of initial treatment. The study was approved by the Institutional Review Board of the Kyoto University Graduate School and Faculty of Medicine Ethics Committee (IRB approval number: G1332).

### 2.2 Statistical Analysis

All data are presented as medians and interquartile ranges (IQRs) or as frequencies and percentages for discrete variables. The Wilcoxon test was used for continuous variables. The Kruskal–Wallis test, followed by Dunn's multiple comparisons test, was used to compare the groups. For the analysis of binary numbers, Pearson's chi‐square test was used to assess the relationships between categorical variables. Given the exploratory nature of this study, no adjustments were made for multiplicity. Statistical significance was defined as a two‐tailed *p*‐value < 0.05. All statistical analyses were performed using JMP Pro 17.0 software (SAS Institute Inc., Cary, NC, US).

## 3 Results

**FIGURE 1:**
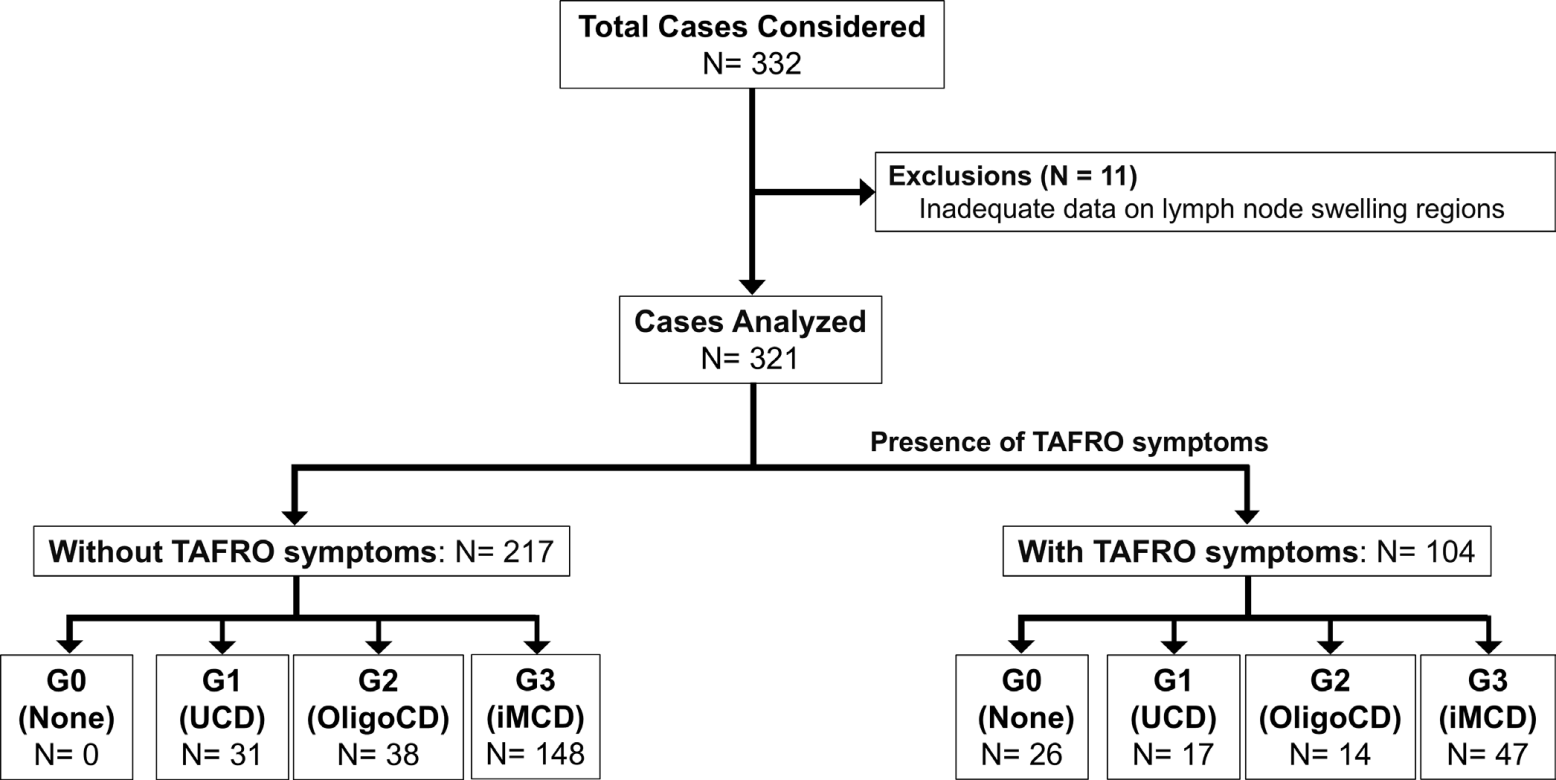
Flowchart of case selection and classification in this study. The total number of cases considered: *N* = 332. Exclusions: *n* = 11 due to inadequate lymph node data or diagnostic inconsistencies. Analyzed cases: *N* = 321. Without TAFRO symptoms: *n* = 217 (unicentric Castleman disease [UCD (G1)]: *n* = 31, oligocentric Castleman disease [OligoCD (G2)]: *n* = 38, idiopathic multicentric Castleman disease [iMCD (G3)]: *n* = 148). TAFRO symptoms, *n* = 104 (UCD (G1), *n* = 17; OligoCD (G2), *n* = 14; iMCD (G3), *n* = 47).

The flowchart of the study is presented in Figure 1. Of the 332 patients reviewed, 11 were excluded because of insufficient data for diagnosis, lack of information on enlarged lymph nodes, or absence of histopathological findings. Of the remaining 321 patients, 217 did not show TAFRO symptoms (118 male patients and 99 female patients), and 104 exhibited TAFRO symptoms (63 male patients, 40 female patients, and 1 patient with unknown gender). Patients with TAFRO syndrome demonstrated significant differences in clinical and laboratory characteristics compared to those without TAFRO syndrome (Table S3). In the absence of TAFRO symptoms, the number of regions involved was high in the G3 group. In the presence of TAFRO symptoms, G0 represented one‐quarter of the cases.

The demographic and laboratory findings of the patients are presented in Table 1. Table 1 illustrates the differences in parameters based solely on the number of lymph node regions, regardless of the presence of TAFRO symptoms. There were 26 patients in G0 (8.1%), 48 in G1 (15%), 52 in G2 (16%), and 195 in G3 (61%). All G0 patients exhibited TAFRO symptoms, with a median C‐reactive protein (CRP) level of 11 mg/dL and a low platelet count of 3.5 × 10^4^/μL. Among the other subtypes (G1–G3), TAFRO symptoms were found in 20%–30%, with the plasmacytic histopathologic subtype dominating (over 60% of the histopathology findings), a median CRP level of > 7 mg/dL, normal median platelet values, and elevated median IgG values.

**TABLE 1:** Histopathological and laboratory parameters in Castleman disease subgroups.

Parameter	G0	G1	G2	G3	All
	*n* = 26 (8.1%)	*n* = 48 (15.0%)	*n* = 52 (16.2%)	*n* = 195 (60.7%)	*N* = 321
Clinical subtype, *n* (%)					
IPL	0 (0.00)	17 (35.4)	20 (38.5)	104 (53.3)	141 (43.9)
NOS	0 (0.00)	14 (29.2)	18 (34.6)	44 (22.6)	76 (23.7)
TAFRO	26 (100)	17 (35.4)	14 (26.9)	47 (24.1)	104 (32.4)
Histopathological subtype, *n* (%)					
Hyaline vascular/hypervascular	0 (0.00)	7 (14.6)	8 (15.4)	23 (11.8)	38 (12.9)
Mixed	0 (0.00)	10 (20.8)	9 (17.3)	18 (9.23)	37 (12.5)
Plasma cell	0 (0.00)	31 (64.6)	35 (67.3)	154 (79.0)	220 (74.6)
CRP (mg/dL), median (IQR) normal range: 0.0–0.14 mg/dL	11.3 (4.97, 22.8)	8.03 (5.13, 15.0)	7.24 (4.35, 10.2)	7.46 (3.77, 12.5)	7.72 (4.1, 13.0)
PLT (×10^4^/μL), median (IQR) normal range: 15.8–34.8 × 10^4^/μL	3.50 (1.45, 6.28)	33.1 (4.7, 41.9)	22 (4.3, 36.9)	31.5 (13.1, 42.4)	27.5 (5.6, 40.5)
IgG (mg/dL), median (IQR) normal range: 861–1747 mg/dL	1279 (879, 1613)	3198 (1723, 4604)	3155 (1974, 4789)	4114 (2221, 5457)	3597 (1677, 5020)

*Note*: G0, patients with TAFRO syndrome without swollen lymph nodes (not CD); G1, CD patients with one lymph node region (UCD); G2, iMCD patients with more than one swollen lymph node region on only one side of the diaphragm (OligoCD); G3, iMCD patients with lesions on the thoracic and abdominal sides of the diaphragm (iMCD).

Abbreviations: CRP, C‐reactive protein; IgG, immunoglobulin G; IPL, idiopathic plasmacytic lymphadenopathy with hyperimmunoglobulinemia; IQR, interquartile range; NOS, not otherwise specified; PLT, platelet count; TAFRO, thrombocytopenia, anasarca, fever, bone marrow fibrosis, and organomegaly.

Table 2 presents the characteristics of the group without TAFRO symptoms and Table 3 presents the characteristics of the group with TAFRO symptoms. As shown in Table 2, the number of cases classified as G3 was the highest (148/217). The overall median age was 49 years, with a slight male predominance. The lymph node swelling sites in G1 and G2 were mostly above the diaphragm (G1, 26/31 cases; G2, 33/38 cases). Clinical subtypes in all groups more frequently indicated IPL than NOS (IPL, 65%; NOS, 35%). The plasmacytic histopathologic subtype was histologically predominant in all groups (G1, 25/31; G2, 32/38; G3, 135/148). Clinical symptoms included inflammatory syndrome and systemic symptoms in approximately 80% of the cases in all groups, pulmonary lesions in 50%–60% of the cases, and hepatosplenomegaly in approximately 40% of the G2 and G3 cases. Laboratory test results and severity findings were similar across groups, with median CRP levels ranging from 6 to 7 mg/dL and high IgG values (median, 4459 mg/dL in all groups; G1, 3888 mg/dL; G2, 4393 mg/dL; G3, 4535 mg/dL).

**TABLE 2:** Clinical and laboratory parameters in Castleman disease subgroups without TAFRO.

TAFRO−	G1	G2	G3	All	
	*n* = 31	*n* = 38	*n* = 148	*N* = 217	*p*
Age, median (IQR)	48.5 (42.5, 61.3)	49 (42.0, 64.0)	49 (39.0, 59.3)	49 (41.0, 61.0)	0.91
Sex, *n* (%)					0.68
Male	19 (61.3)	21 (55.3)	78 (52.7)	118 (54.4)	
Female	12 (38.7)	17 (44.7)	70 (47.3)	99 (45.6)	
Side of diaphragm, *n*					
Above	26 (83.9)	33 (86.8)	0 (0.00)	59 (27.2)	
Below	5 (16.1)	5 (13.2)	0 (0.00)	10 (4.6)	
Full body	0 (0.00)	0 (0.00)	148 (100)	148 (68.2)	
Region, *n* (%)					
Head/neck	13 (41.9)	23 (60.5)	117 (79.1)	153 (70.5)	< 0.00010^*^
Mediastinal/hilar	10 (32.3)	16 (42.1)	92 (64.3)	118 (55.7)	0.00090^*^
Right axillary	2 (6.45)	26 (68.4)	124 (83.8)	152 (70.1)	< 0.00010^*^
Left axillary	1 (3.23)	23 (60.5)	123 (83.1)	147 (67.7)	< 0.00010^*^
Abdominal	4 (12.9)	3 (7.89)	84 (57.5)	91 (42.3)	< 0.00010^*^
Right inguinal	1 (3.23)	5 (13.2)	127 (85.8)	133 (61.3)	< 0.00010^*^
Left inguinal	0 (0.00)	5 (13.2)	129 (87.2)	134 (61.8)	< 0.00010^*^
Clinical subtype, *n* (%)					0.056
IPL	17 (54.8)	20 (52.6)	104 (70.3)	141 (65.0)	
NOS	14 (45.2)	18 (47.4)	44 (29.7)	76 (35.0)	
Inflammatory syndrome, *n* (%)	26 (83.9)	35 (92.1)	137 (92.6)	198 (91.2)	0.29
Histopathological subtype, *n* (%)					0.036
Hyaline vascular /Hypervascular	2 (6.45)	1 (2.63)	3 (2.03)	6 (2.76)	
Mixed	4 (12.9)	5 (13.2)	10 (6.76)	19 (8.76)	
Plasma cell	25 (80.7)	32 (84.2)	135 (91.2)	192 (88.5)	
Clinical symptoms, *n* (% of those assessed)					
Constitutional symptoms	26 (83.9)	29 (80.6)	129 (87.2)	184 (85.6)	0.57
Hepatosplenomegaly	5 (18.5)	12 (38.7)	56 (44.8)	73 (39.9)	0.043^*^
Cherry haemangioma/violaceous papules	3 (12.0)	7 (30.4)	38 (34.6)	48 (30.4)	0.087
Interstitial lung disease (ILD)	7 (53.9)	9 (56.3)	54 (65.1)	70 (62.5)	0.63
Fluid retention	4 (14.8)	7 (20.6)	21 (15.2)	32 (16.1)	0.73
CRP (mg/dL), median (IQR) normal range: 0.0–0.14 mg/dL	7.56 (5.04, 12.0)	6.00 (3.40, 8.13)	6.48 (3.47, 10.9)	6.57 (3.76, 10.4)	0.010
PLT (×10^4^/μL), median (IQR) normal range: 15.8–34.8 × 10^4^/μL	39.0 (33.1, 47.0)	30.3 (18.3, 40.5)	35.7 (27.2, 44.3)	35.7 (26.4, 44.2)	0.049^*^
Hb (g/dL), median (IQR) normal range: 13.7–16.8 g/dL (male), 11.6–14.8 g/dL (female)	10.6 (9.20, 11.6)	10.1 (8.08, 11.3)	10.0 (8.40, 11.8)	10.1 (8.40, 11.6)	0.76
Alb (g/dL), median (IQR) normal range: 4.1–5.1 g/dL	2.9 (2.40, 3.30)	2.70 (2.50, 3.30)	2.70 (2.20, 3.30)	2.78 (2.30, 3.30)	0.70
Cr (mg/dL), median (IQR) normal range: 0.65–1.07 g/dL (male), 0.46–0.79 g/dL (female)	0.710 (0.600, 0.900)	0.730 (0.600, 1.12)	0.750 (0.600, 0.940)	0.730 (0.600, 0.955)	0.57
eGFR (mL/min/1.73m^2^), *n* (% of those assessed)					0.42
60+	14 (82.4)	19 (76.0)	69 (73.4)	102 (75.0)	
30–60	3 (17.7)	3 (12.0)	21 (22.3)	27 (19.9)	
15–30	0 (0.00)	0 (0.00)	1 (1.06)	1 (0.735)	
0–15 or requires hemodialysis	0 (0.00)	3 (12.0)	3 (3.19)	6 (4.41)	
ALP (IU/mL), median (IQR) normal range: 106–322 U/L	273 (225, 378)	269 (213, 308)	268 (200, 334)	269 (208, 333)	0.81
IgA (mg/dL), median (IQR) normal range: 93–393 mg/dL	618 (360, 870)	505 (342, 659)	543 (405, 710)	540 (382, 711)	0.44
IgM (mg/dL), median (IQR) normal range: 33–187 mg/dL (male), 50–269 mg/dL (female)	187 (137, 272)	202 (92, 261)	224 (146, 334)	217 (137, 312)	0.21
IgG (mg/dL), median (IQR) normal range: 861–1747 mg/dL	3888 (3176, 5381)	4393 (2981, 5111)	4535 (3653, 5764)	4459 (3307, 5632)	0.19
IgG4 (mg/dL), median (IQR) normal range: 11–121 mg/dL	335 (69.4, 634)	309 (175, 653)	398 (204, 863)	338 (176, 795)	0.49
IgE (IU/mL), median (IQR) normal range: 0–358 IU/mL	1096 (141, 1666)	420 (82.5, 2878)	1172 (388, 3673)	1137 (308, 3270)	0.31
CHAP score, median (IQR)	6.00 (4.00, 9.00)	6.00 (4.00, 8.00)	6.00 (3.00, 8.00)	6.00 (4.00, 9.00)	0.94

*Note*: G0: patients with TAFRO syndrome without swollen lymph nodes (not CD); G1: CD patients with one lymph node region (UCD); G2: iMCD patients with more than one swollen lymph node region on only one side of the diaphragm (OligoCD); G3: iMCD patients with lesions on the thoracic and abdominal sides of the diaphragm (iMCD). *p*‐values were calculated to evaluate statistical significance between groups. Continuous variables were compared using the Kruskal–Wallis test, and categorical variables were analyzed using the Pearson's chi‐square test. A *p*‐value of < 0.05 was considered statistically significant.

* indicates that these differences between the groups (G0‐G3) were statistically significant at the *p* < 0.05 level.

Abbreviations: Alb, albumin; ALP, alkaline phosphatase; Cr, creatinine; CRP, C‐reactive protein; eGFR, estimated glomerular filtration rate; Hb, hemoglobin; IgA, immunoglobulin A; IgE, immunoglobulin E; IgG, immunoglobulin G; IgM, immunoglobulin M; ILD, interstitial lung disease; iMCD, idiopathic multicentric Castleman disease; IPL, idiopathic plasmacytic lymphadenopathy with hyperimunnogloblinemia; IQR, interquartile range; NOS, not otherwise specified; PLT, platelet count; TAFRO, thrombocytopenia, anasarca, fever, bone marrow fibrosis, and organomegaly.

**TABLE 3:** Clinical and laboratory parameters in Castleman disease subgroups with TAFRO.

TAFRO+	G0	G1	G2	G3	All	
	*n* = 26	*n* = 17	*n* = 14	*n* = 47	*N* = 104	*p*
Age, median (IQR)	55 (44.0, 69.0)	52.5 (46.3, 65.5)	64 (58.0, 70.5)	48 (40.0, 68.0)	53 (44.0, 66.0)	0.023^*^
Sex, *n* (%)						0.0020^*^
Male	11 (42.3)	6 (35.3)	10 (76.9)	36 (76.6)	63 (61.2)	
Female	15 (57.7)	11 (64.7)	3 (23.1)	11 (23.4)	40 (38.8)	
Side of diaphragm, *n*						
Above	0 (0.00)	16 (94.1)	11 (78.6)	0 (0.00)	27 (34.6)	
Below	0 (0.00)	1 (5.9)	3 (21.4)	0 (0.00)	4 (5.1)	
Full body	0 (0.00)	0 (0.00)	0 (0.00)	47 (100)	47 (60.3)	
Region, *n* (%)						
Head/neck	0 (0.00)	12 (70.6)	7 (50.0)	38 (82.6)	57 (55.3)	< 0.00010^*^
Mediastinal/hilar	0 (0.00)	1 (5.88)	7 (50.0)	26 (55.3)	34 (32.7)	< 0.00010^*^
Right axillary	0 (0.00)	3 (17.7)	8 (57.1)	35 (74.4)	46 (44.2)	< 0.00010^*^
Left axillary	0 (0.00)	0 (0.00)	6 (42.9)	37 (78.7)	43 (41.4)	< 0.00010^*^
Abdominal	0 (0.00)	0 (0.00)	2 (14.3)	33 (70.2)	35 (33.7)	< 0.00010^*^
Right inguinal	0 (0.00)	0 (0.00)	3 (21.4)	31 (66.0)	34 (32.7)	< 0.00010^*^
Left inguinal	0 (0.00)	1 (5.88)	2 (15.4)	31 (66.0)	34 (33.0)	< 0.00010^*^
Inflammatory syndrome, *n* (%)	25 (96.1)	15 (100)	13 (92.9)	42 (95.5)	95 (96.0)	0.80
Histopathological subtype, *n* (%)						0.41
Hyaline vascular /hypervascular	0 (0.00)	5 (29.4)	7 (50.0)	20 (42.6)	32 (41.0)	
Mixed	0 (0.00)	6 (35.3)	4 (28.6)	8 (17.0)	18 (23.1)	
Plasma cell	0 (0.00)	6 (35.3)	3 (21.4)	19 (40.4)	28 (35.9)	
Clinical symptoms, *n* (% of those assessed)						
Constitutional symptoms	26 (100)	16 (94.1)	14 (100)	41 (91.1)	97 (95.1)	0.30
Hepatosplenomegaly	9 (36.0)	13 (76.5)	9 (64.3)	28 (60.9)	59 (57.8)	0.17
Cherry haemangioma/violaceous papules	6 (25.0)	2 (13.3)	1 (10.0)	5 (14.7)	14 (16.9)	0.63
Lung disease (ILD)	0 (0.00)	0 (0.00)	0 (0.00)	2 (12.5)	2 (9.09)	0.662
Fluid retention	26 (100)	17 (100)	14 (100)	46 (100)	103 (100)	—
CRP (mg/dL), median (IQR) normal range: 0.0–0.14 mg/dL	11.3 (4.97, 22.8)	13 0.6 (5.18, 19.1)	14.2 (8.28, 22.6)	14.3 (7.14, 22.8)	14.15 (6.59, 21.6)	0.94
PLT (×10^4^/μL), median (IQR) normal range: 15.8–34.8 × 10^4^/μL	3.5 (1.45, 6.28)	2.75 (1.25, 4.85)	3.00 (1.55, 4.15)	4.85 (2.21, 8.80)	3.55 (1.73, 5.85)	0.078
Hb (g/dL), median (IQR) normal range: 13.7–16.8 g/dL (male), 11.6–14.8 g/dL (female)	9.05 (7.48, 11.1)	9.10 (7.40, 10.2)	9.50 (7.4, 10.7)	10.3 (8.35, 12.5)	9.6 (8.05, 11.4)	0.25
Alb (g/dL), median (IQR) normal range: 4.1–5.1 g/dL	2.28 (1.97, 2.63)	2.10 (1.30, 2.50)	2.40 (1.90, 2.80)	2.20 (1.67, 2.70)	2.20 (1.80, 2.70)	0.62
Cr (mg/dL), median (IQR) normal range: 0.65–1.07 g/dL (male), 0.46–0.79 g/dL (female)	1.70 (0.905, 2.03)	1.53 (1.18, 2.56)	1.66 (1.02, 2.88)	1.69 (1.18, 2.67)	1.66 (1.14, 2.49)	0.95
eGFR (mL/min/1.73m^2^), *n* (% of those assessed)						0.25
60+	0 (0.00)	1 (12.5)	1 (12.5)	5 (17.2)	7 (12.3)	
30–60	2 (16.7)	5 (62.5)	3 (37.5)	10 (34.5)	20 (35.1)	
15–30	3 (25.0)	1 (12.5)	3 (37.5)	7 (24.1)	14 (24.6)	
0–15 or requires hemodialysis	7 (58.3)	1 (12.5)	1 (12.5)	7 (24.1)	16 (28.1)	
ALP (IU/mL), median (IQR) normal range: 106–322 U/L	503 (383, 1026)	414 (267, 814)	614 (349, 1032)	498 (348, 846)	498 (353, 847)	0.49
IgA (mg/dL), median (IQR) normal range: 93–393 mg/dL	204 (159, 272)	222 (173, 299)	196 (156, 266)	173 (138, 210)	191 (151, 246)	0.093
IgM (mg/dL), median (IQR) normal range: 33–187 mg/dL (male), 50–269 mg/dL (female)	77 (49, 143)	89 (65, 115)	101 (55, 190)	70 (52, 89)	79 (54, 105)	0.13
IgG (mg/dL), median (IQR) normal range: 861–1747 mg/dL	1279 (879, 1613)	1504 (1176, 1751)	1176 (861, 1766)	1264 (895, 1672)	1299 (918.0, 1631)	0.45
IgG4 (mg/dL), median (IQR) normal range: 11–121 mg/dL	16.0 (13.1, 36.0)	34.7 (20.8, 95.5)	44.6 (29.3, 78.1)	38.0 (18.0, 81.5)	32.5 (17.2, 74.8)	0.11
IgE (IU/mL), median (IQR) normal range: 0–358 IU/mL	67.8 (31.4, 70.9)	50.4 (13.0, 188)	170 (25.0, 837)	102 (40.5, 367)	70.3 (37.4, 249)	0.49
CHAP score, median (IQR)	9.00 (6.00, 11.0)	11.0 (7.00, 11.0)	8.00 (7.00, 11.5)	8.50 (6.75, 10.0)	9.00 (7.00, 11.0)	0.61
Severity score for TAFRO syndrome, median (IQR)	9.00 (8.00, 10.0)	8.50 (7.25, 9.00)	8.00 (7.00, 9.75)	8.00 (7.00, 9.00)	8.00 (8.00, 10.0)	0.39
Severity grade for TAFRO syndrome, *n* (%)						0.90
Grade 1	0 (0.00)	0 (0.00)	0 (0.00)	1 (3.9)	1 (1.9)	
Grade 2	0 (0.00)	0 (0.00)	0 (0.00)	3 (11.5)	3 (5.7)	
Grade 3	4 (36.4)	4 (44.4)	4 (57.1)	11 (42.3)	23 (43.4)	
Grade 4	5 (45.5)	3 (33.3)	2 (28.6)	9 (34.6)	19 (35.9)	
Grade 5	2 (18.2)	2 (22.2)	1 (14.3)	2 (7.7)	7 (13.2)	

*Note*: G0: patients with TAFRO syndrome without swollen lymph nodes (not CD); G1: CD patients with one lymph node region (UCD); G2: iMCD patients with more than one swollen lymph node region on only one side of the diaphragm (OligoCD); G3: iMCD patients with lesions on the thoracic and abdominal sides of the diaphragm (iMCD). *p*‐values were calculated to evaluate statistical significance between groups. Continuous variables were compared using the Kruskal‐Wallis test, and categorical variables were analyzed using the Pearson's chi‐squared test. A *p*‐value of < 0.05 was considered statistically significant.

* indicates that these differences between the groups (G0‐G3) were statistically significant at the *p* < 0.05 level.

Abbreviations: Alb, albumin; ALP, alkaline phosphatase; Cr, creatinine; CRP, C‐reactive protein; eGFR, estimated glomerular filtration rate; Hb, hemoglobin; IgA, immunoglobulin A; IgE, immunoglobulin E; IgG, immunoglobulin G; IgM, immunoglobulin M; ILD, interstitial lung disease; IPL, idiopathic plasmacytic lymphadenopathy with hyperimunnogloblinemia; IQR, interquartile range; NOS, not otherwise specified; PLT, platelet count; TAFRO, thrombocytopenia, anasarca, fever, bone marrow fibrosis, and organomegaly.

**FIGURE 2:**
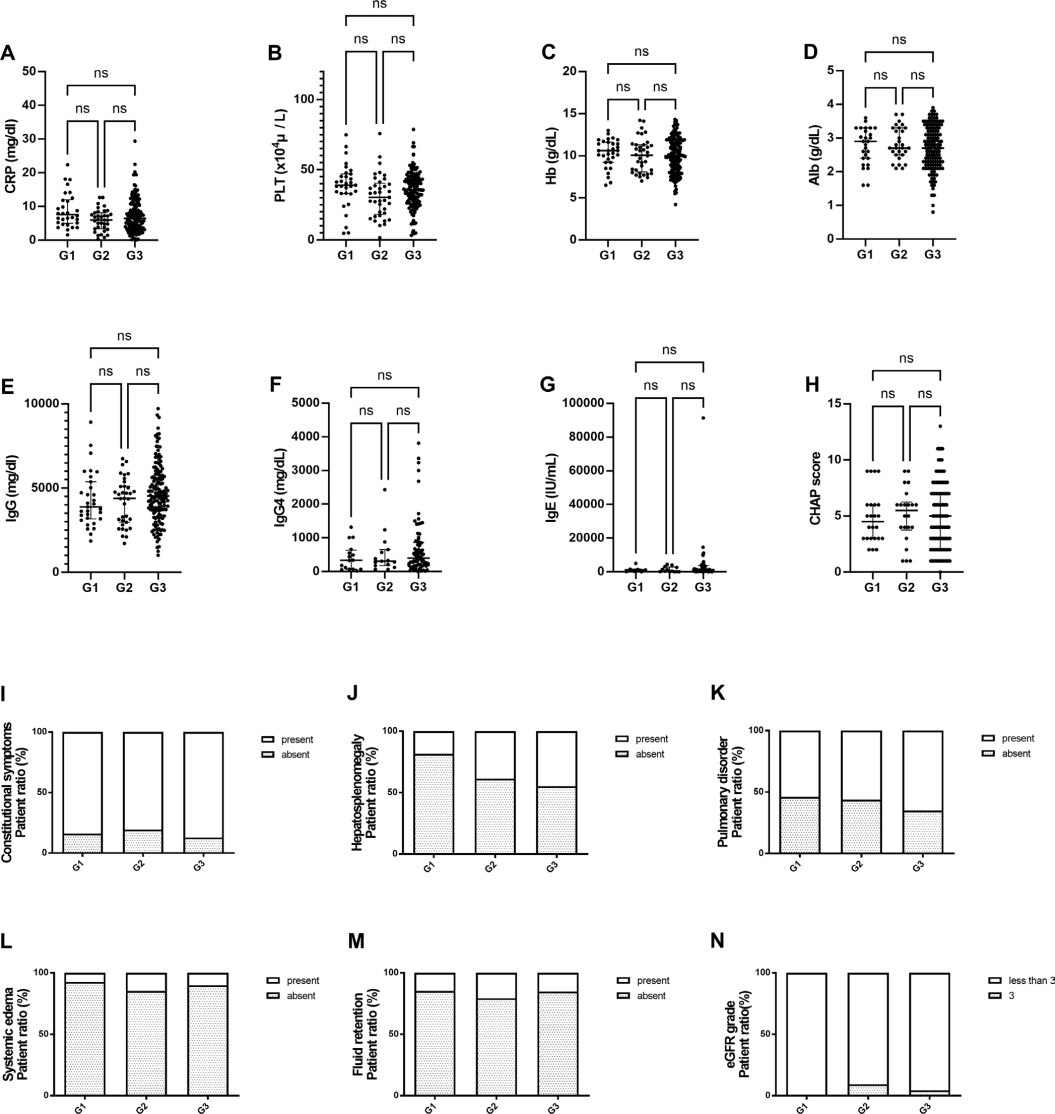
Comparative analysis in cases without TAFRO symptoms according to lymph node region counts. Scatter plots (A–H) and bar graphs (I–N) display various laboratory parameters across different lymph node region count types in cases without TAFRO symptoms: C‐reactive protein (CRP) level (A), platelet count (PLT) (B), hemoglobin (Hb) level (C), albumin (Alb) level (D), immunoglobulin (Ig) G (IgG) level (E), IgG4 level (F), IgE level (G), and CHAP score (H). Clinical features are presented as I–N: Constitutional symptoms (I), hepatosplenomegaly (J), pulmonary disorder (K), systemic edema (L), fluid retention (M), and eGFR grade (N). “ns” indicates no significant difference between groups. G0: Patients with TAFRO syndrome without swollen lymph nodes (not CD); G1: CD patients with one lymph node region (UCD); G2: IMCD patients with more than one swollen lymph node region on only one side of the diaphragm (OligoCD); G3: IMCD patients with lesions on the thoracic and abdominal sides of the diaphragm (iMCD).

In the group without TAFRO symptoms, analysis according to the number of enlarged lymph node regions did not reveal any significant differences in laboratory values or CHAP scores (Figure 2A–H). Figure 2I–N displays the prevalence of constitutional symptoms, hepatomegaly, pulmonary disorders, systemic edema, fluid retention, and estimated glomerular filtration rate across the three patient groups (G1–G3). Importantly, there was a significant difference in the incidence of hepatosplenomegaly (Figure 2J, *p* = 0.043), which increased with an increase in the number of swollen regions.

Even in the presence of TAFRO symptoms (Table 3), the number of cases classified as G3 was the highest (47/104 cases). The overall median age was 53 years, with a high male ratio among cases with TAFRO symptoms, and lymph node swelling sites in G1 and G2 were more common above the diaphragm (G1:16/17, G2:11/14). In patients with iMCD‐TAFRO (G1–G3) the hypervascular pathological type was the most common. Inflammatory syndrome and systemic symptoms were observed in approximately 90% of the cases in all groups. Moreover, pulmonary lesions were rare, and the overall median CRP value exceeded 10 mg/dL with elevated creatinine levels. The median IgG values were within the normal range (median: 1299 mg/dL in all groups).

**FIGURE 3:**
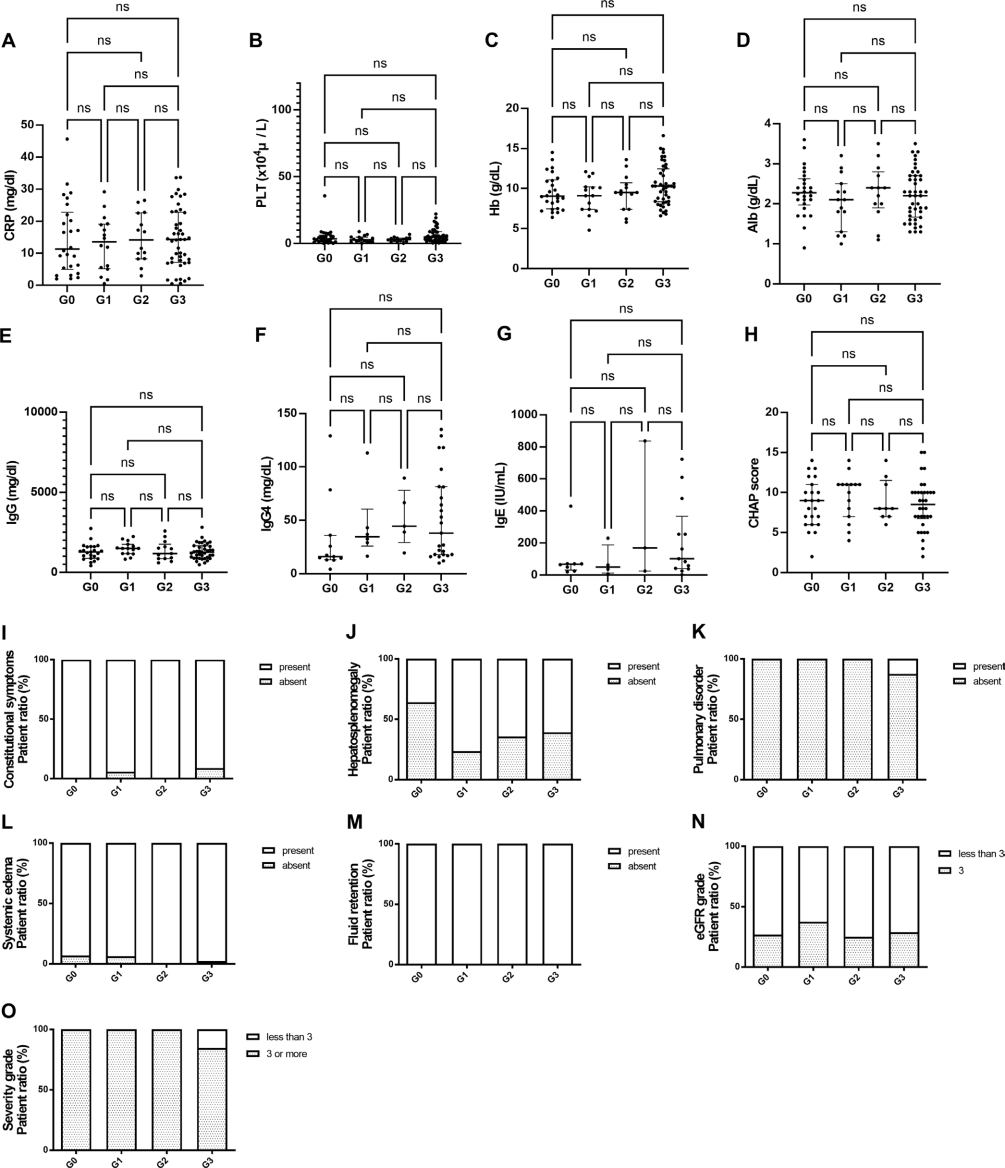
Comparative analysis in cases with TAFRO symptoms according to lymph node region counts. Scatter plots (A–H) and bar graphs (I–O) display various laboratory parameters across different lymph node region count types in cases with TAFRO symptoms: C‐reactive protein (CRP) level (A), platelet count (PLT) (B), hemoglobin (Hb) level (C), albumin (Alb) level (D), immunoglobulin (Ig) G (IgG) level (E), IgG4 level (F), IgE level (G), and CHAP score (H). Clinical features are presented in I–N: Constitutional symptoms (I), hepatosplenomegaly (J), pulmonary disorder (K), systemic edema (L), fluid retention (M), and eGFR grade (N). Severity, categorized as less than three swollen lymph node regions or three or more regions, is shown in (O). “ns” indicates no significant difference between groups. G0: Patients with TAFRO syndrome without swollen lymph nodes (not CD); G1: CD patients with one lymph node region (UCD); G2: IMCD patients with more than one swollen lymph node region on only one side of the diaphragm (OligoCD); G3: IMCD patients with lesions on the thoracic and abdominal sides of the diaphragm (iMCD).

In the group with TAFRO symptoms, the analysis according to the number of enlarged lymph node regions showed no significant differences in laboratory values or CHAP scores (Figure 3A–H). Figure 3I–N displays the prevalence of constitutional symptoms, hepatomegaly, pulmonary disorders, systemic edema, fluid retention, and estimated glomerular filtration rate across the four patient groups (G0–G3). There were no significant differences in the distribution of these clinical features among the groups, indicating that these symptoms and conditions were present uniformly across all categories. When severity was compared according to the TAFRO syndrome severity classification 2019 [19], no significant difference was noted (Figure 3O).

## 4 Discussion

In this study, we used data from a multicenter cohort in Japan to retrospectively analyze whether CD presents with different characteristics depending on the distribution of enlarged lymph node regions. In the study by Pierson et al. [14], 25% of patients were categorized as having UCD (corresponding to G1 in this study), in contrast to our study, where only 15% of cases were identified as G1. Pierson et al. reported no patients with UCD (G1 in this study) with TAFRO symptoms and only one patient with OligoCD (corresponding to G2 in this study) with these symptoms, whereas 61% of patients with iMCD (corresponding to G3 in this study) presented with TAFRO symptoms. Unlike their findings, our study observed that TAFRO symptoms were uniformly distributed among different categories. Furthermore, their report shows that the histological types of UCD, OligoCD, and iMCD are mainly hyaline vascular/ hypervascular types, accounting for 93%, 75%, and 68%, respectively. On the other hand, our study shows that the percentage of hyaline vascular/ hypervascular types is low in G1–G3 cases, while more than 60% of cases are plasmacytic histopathologic subtype. Regarding UCD, previous reports suggest that both Japan and the United States tend to have a high prevalence of the hyaline vascular type, with approximately 75% in both populations [20, 21]. Thus, the differences observed in this study are unlikely to be due to ethnic, geographic, or environmental factors but are more likely attributable to methodological differences.

Regarding median CRP levels, the report by Pierson et al. indicated that for UCD and OligoCD, the levels were low, at 1–2 mg/dL, while for iMCD, they were higher, at 9 mg/dL. In our study, however, G1–G3 exhibited elevated CRP levels ranging between 7 and 8 mg/dL. As for median IgG values, Pierson et al. reported that UCD had a level of 1049 mg/dL and OligoCD 1451 mg/dL, both within normal ranges, whereas iMCD showed a slightly elevated level of 1868 mg/dL. In contrast, our findings showed significantly higher IgG levels in G1–G3, ranging between 3000 and 4000 mg/dL across all groups, including those with and without TAFRO symptoms.

The results of this study are consistent with previous reports [22, 23], potentially indicating that the relative frequency of UCD to MCD in Japan is not as high as that in the United States. The discrepancies in the incidence rates of UCD between the studies can be partially attributed to differences in the inclusion criteria. Our study predominantly registered patients who presented to the internal medicine department with systemic symptoms, with very few cases involving asymptomatic lymph node enlargement. This led to a higher proportion of systemic and inflammatory symptoms. In contrast, the American study [14] utilized a patient registry approach that included all CD cases regardless of the presence of symptoms or inflammation. This typically encompassed many classic UCD cases that do not involve systemic inflammation or IL‐6 production and are histologically identified as hyaline vascular type [13, 20, 24]. These differences in case selection might explain the higher levels of inflammatory markers and systemic symptoms observed in our cohort.

TAFRO syndrome is generally recognized as occurring without lymph node swelling or in association with iMCD. However, our study revealed that cases of iMCD‐TAFRO are not limited to G3 but also exist significantly in G1 and G2. This is a notable finding because a significant number of patients with UCD who exhibit inflammatory symptoms and signs also show hyaline vascular/hypervascular lymph node pathology. To our knowledge, such cases have not been reported in the literature before. In this study, we did not differentiate histologically between hypervascular and hyaline vascular types, leaving the specifics unclear; however, it is plausible that most G1 cases exhibiting TAFRO symptoms are of the hypervascular type. A detailed pathological analysis is necessary in the future. It cannot be ruled out that the cases were categorized as G1 or G2 due to their initial states or because they were unrecognized on CT imaging. Nevertheless, the finding that patients with TAFRO symptoms can present with various distributions of enlarged lymph node counts is a significant outcome of this study and warrants further verification.

In the group without TAFRO symptoms, an increase in the number of swollen lymph node regions was correlated with an increase in the incidence of hepatosplenomegaly. No other significant differences were observed between groups. A large number of swollen lymph node regions did not necessarily indicate greater severity. Although crossing the diaphragm is considered a poor prognostic factor in malignant lymphoma [15], our analysis showed no difference in outcomes for iMCD regardless of whether the diaphragm was crossed. The lack of significant differences in the number and distribution of swollen lymph node regions suggests that swollen lymph nodes alone do not capture the full picture of the disease. When comparing the group with TAFRO symptoms in this study to that in the report by Kawabata et al. [25], our group had a slightly higher ratio of male patients, but there were no significant differences in age, proportion of accompanying iMCD, or median laboratory values.

Extramedullary hematopoiesis (EMH) has been reported in iMCD, particularly in the TAFRO subtype, as demonstrated in a recent case series [26]. This study described four cases of iMCD with EMH identified in lymph node biopsies, including two with hypervascular histology and two with mixed histology. However, it is notable that no cases of EMH were identified in our study cohort of iMCD‐TAFRO or TAFRO syndrome patients. This discrepancy may reflect differences in cohort size, diagnostic methods, or population characteristics, and further studies are needed to explore the prevalence and significance of EMH in TAFRO syndrome.

An emerging concept in Castleman disease is asymptomatic multicentric Castleman disease (aMCD), which describes HHV‐8‐negative MCD patients who lack symptoms and a hyperinflammatory state, not meeting the criteria for iMCD. A retrospective study of 114 aMCD patients reported that some cases transformed into iMCD over time [27]. In our cohort, although about 10% of cases lacked a clear inflammatory syndrome, nearly all showed elevated inflammatory markers, including CRP. This indicates that aMCD cases are likely underrepresented in our study population. Further research is needed to examine the number and distribution of lymph node regions in aMCD cases to better understand their clinical significance and potential progression.

In our study, we observed that interstitial lung disease was more common in patients without TAFRO signs, while the incidence of interstitial lung disease was lower in patients with TAFRO signs, regardless of the number of affected lymph node regions. As TAFRO syndrome generally has an acute onset, this may explain why the incidence of chronic inflammatory diseases such as interstitial lung disease is generally lower than that of iMCD, which progresses more slowly. Interestingly, ILD is generally more common in patients of East Asian descent, including Japanese, and is rarely observed in White patients. The ethnic and disease‐specific variations in ILD symptoms in iMCD have not been emphasized in previous literature, and may indicate potential genetic or environmental factors that affect the pathogenesis of the disease.

The present study has several limitations. This study was retrospective in nature, which may introduce biases such as selection bias or incomplete records; however, given the rarity of the disease, conducting a retrospective study was unavoidable. The small number of cases necessitates future data accumulation and analysis. Severely ill patients may die without being diagnosed or evaluated in internal medicine clinicals needed for enrollment. Furthermore, pathological diagnoses and classifications of lymph nodes were determined by individual facilities, which could have impacted consistency. These factors collectively underline the need for a cautious interpretation of our findings, and subsequent studies are strongly recommended to address these limitations.

## 5 Conclusion

In this study, we analyzed whether CD exhibits distinct characteristics based on the distribution of lymph node regions involved. Contrary to reports from the United States, in Japan, systemic inflammation was uncommon in both UCD and iMCD cases. The proportion of cases exhibiting TAFRO symptoms was consistent across all types as defined by the number and distribution of lymph node regions. Regardless of the presence or absence of TAFRO symptoms, it was challenging to discern clinical characteristics based solely on the number of enlarged lymph node regions in CD cases.

## Author Contributions

T.K. and A.K. were responsible for conceptualizing the study, developing the methodology, supervising the project, and acquiring funding. R.S., S.F., Y.K., T.S., S.Y., Y.M., M.I., H.Y., M.K., Y.S., N.S., and H.N. provided the resources needed for the execution of the study and assisted in the data curation and revision of the manuscript. M.O. and T.K. analyzed the data and wrote the manuscript. All authors have read and approved the final manuscript, ensuring the accuracy and integrity of the work.

## Conflicts of Interest

The authors declare no conflicts of interest.

## Supporting information


**Table S1.** CHAP Score for Assessing the Severity of iMCD.

**Table S2.** Diagnostic Criteria for TAFRO Syndrome, according to the Revised Guidelines 2015.

**Table S3.** Clinical and laboratory characteristics of patients with and without TAFRO syndrome.

## Data Availability

The datasets generated and analyzed during the current study are not publicly available because of privacy or ethical restrictions but are available from the corresponding author upon reasonable request.
