# Supplementary material for: Exploring the Clinical Diversity of Castleman Disease and TAFRO Syndrome: A Japanese Multicenter Study on Lymph Node Distribution Patterns
Source: Am J Hematol. 2025 Jan 25;100(4):592–605. doi: 10.1002/ajh.27612 (PMC11886485; doi:10.1002/ajh.27612)
Supplement: Supplementary file 1 — Table S1. CHAP Score for Assessing the Severity of iMCD. [file AJH-100-592-s001.docx]

**Supplementary Table S1: CHAP Score for Assessing the Severity of iMCD**

| **Score** | **1** | **2** | **3** | **4** |
| --- | --- | --- | --- | --- |
| CRP (mg/dL) | ≥1, <5 | ≥5, <10 | ≥10, <20 | ≥20 |
| Hb (g/dL) | <12, ≥10 | <10, ≥8 | <8 |  |
| Alb (g/dL) | <3, ≥2.5 | <2.5, ≥2 | <2, ≥1.5 | <1.5 |
| PS | 1 | 2 | 3 | 4 |

This score incorporates clinical laboratory values including C-reactive protein (CRP), hemoglobin (Hb), and albumin (Alb), along with the patient's performance status (PS). The scoring ranges for each parameter are as the table above. The score increases with higher CRP levels, lower hemoglobin, and albumin levels, as well as deteriorating patient performance status, indicating more severe inflammation.
