# Supplementary material for: Exploring the Clinical Diversity of Castleman Disease and TAFRO Syndrome: A Japanese Multicenter Study on Lymph Node Distribution Patterns
Source: Am J Hematol. 2025 Jan 25;100(4):592–605. doi: 10.1002/ajh.27612 (PMC11886485; doi:10.1002/ajh.27612)
Supplement: Supplementary file 2 — Table S2. Diagnostic Criteria for TAFRO Syndrome, according to the Revised Guidelines 2015. [file AJH-100-592-s003.docx]

**Supplementary Table S2: Diagnostic Criteria for TAFRO Syndrome, according to the Revised Guidelines 2015**

| **Symptom or Condition** | **Points** |
| --- | --- |
| **Anasarca** |  |
| - Pleural effusion on imaging | 1 point |
| - Ascites on imaging | 1 point |
| - Pitting edema on physical examination | 1 point |
| **Thrombocytopenia** |  |
| - Platelet counts <100,000/μl | 1 point |
| - Platelet counts <50,000/μl | 2 points |
| - Platelet counts <10,000/μl | 3 points |
| **Fever and/or Inflammation** |  |
| - Fever ≥37.5°C but <38.0°C or CRP ≥2 mg/dl but <10 mg/dl | 1 point |
| - Fever ≥38.0°C but <39.0°C or CRP ≥10 mg/dl but <20 mg/dl | 2 points |
| - Fever ≥39.0°C or CRP ≥20 mg/dl | 3 points |
| **Renal Insufficiency** |  |
| - GFR <60 ml/min/1.73 m² | 1 point |
| - GFR <30 ml/min/1.73 m² | 2 points |
| - GFR <15 ml/min/1.73 m² or need for hemodialysis | 3 points |
| Relationship between score and disease severity | |
| **Total Points** | **Severity Classification** |
| 0–2 points | Insufficient for diagnosis |
| 3–4 points | Mild (grade 1) |
| 5–6 points | Moderate (grade 2) |
| 7–8 points | Slightly severe (grade 3) |
| 9–10 points | Severe (grade 4) |
| 11–12 points | Very severe (grade 5) |

These criteria encompass a range of clinical and laboratory features, scored to assess the severity and presence of the syndrome.
