# Supplementary material for: Exploring the Clinical Diversity of Castleman Disease and TAFRO Syndrome: A Japanese Multicenter Study on Lymph Node Distribution Patterns
Source: Am J Hematol. 2025 Jan 25;100(4):592–605. doi: 10.1002/ajh.27612 (PMC11886485; doi:10.1002/ajh.27612)
Supplement: Supplementary file 3 — Table S3. Clinical and laboratory characteristics of patients with and without TAFRO syndrome. [file AJH-100-592-s002.docx]

**Supplementary table S3. Clinical and laboratory characteristics of patients with and without TAFRO syndrome.**

|  | **All** | **without TAFRO** | **with TAFRO** | **P value** |
| --- | --- | --- | --- | --- |
|  | N = 321 | n = 217 | n = 104 |  |
| **Age,** median (IQR) | 49 (42.0, 63.0) | 49 (41.0, 61.0) | 53 (44.0, 66.0) | 0.050 |
| **Sex**, n (%) |  |  |  | 0.25 |
| Male | 181 (56.6) | 118 (54.4) | 63 (61.2) |  |
| Female | 139 (43.4) | 99 (45.6) | 40 (38.8) |  |
| **Side of diaphragm**, n (%) |  |  |  |  |
| Above | 86 (29.2) | 59 (27.2) | 27 (34.6) |  |
| Below | 14 (4.70) | 10 (4.60) | 4 (5.10) |  |
| Full body | 195 (66.1) | 148 (68.2) | 47 (60.3) |  |
| **Region**, n (%) |  |  |  |  |
| Head/neck | 210 (65.6) | 153 (70.5) | 57 (55.3) | 0.0076* |
| Mediastinal/hilar | 152 (48.1) | 118 (55.7) | 34 (32.7) | <0.00010* |
| Right axillary | 198 (61.7) | 152 (70.1) | 46 (44.2) | <0.00010* |
| Left axillary | 190 (59.2) | 147 (67.7) | 43 (41.4) | <0.00010* |
| Abdominal | 126 (39.5) | 91 (42.3) | 35 (33.7) | 0.14 |
| Right inguinal | 167(52.0) | 133 (61.3) | 34 (32.7) | <0.00010* |
| Left inguinal | 168 (52.5) | 134 (61.8) | 34 (33.0) | <0.00010* |
| **Inflammatory syndrome**, n (%) | 279(91.2) | 184 (88.9) | 95 (96.0) | 0.041* |
| **Histopathological subtype**, n (%) |  |  |  | <0.00010* |
| Hyaline vascular /Hypervascular | 38 (12.9) | 6 (2.76) | 32 (41.0) |  |
| Mixed | 37 (12.5) | 19 (8.76) | 18 (23.1) |  |
| Plasma cell | 220 (74.6) | 192 (88.5) | 28 (35.9) |  |
| **Clinical symptoms**, n (% of those assessed) |  |  |  |  |
| Constitutional symptoms | 281 (88.6) | 184 (85.6) | 97 (95.1) | 0.30 |
| Hepatosplenomegaly | 132 (46.3) | 73 (39.9) | 59 (57.8) | 0.0036* |
| Cherry haemangioma/violaceous papules | 62 (25.7) | 48 (30.4) | 14 (16.9) | 0.023* |
| Lung disease (ILD) | 72 (53.7) | 70 (62.5) | 2 (9.09) | <0.00010* |
| Fluid retention | 135 (44.7) | 32 (16.1) | 103 (100) | <0.00010* |
| **CRP** (mg/dL), median (IQR)  normal range: 0.0-0.14 mg/dL | 7.72 (4.10, 13.0) | 6.57 (3.76, 10.4) | 14.15 (6.59, 21.6) | <0.00010* |
| **PLT** (×10^4^/μL), median (IQR)  normal range: 15.8-34.8 × 10⁴/µL | 27.5 (5.60, 40.5) | 35.7 (26.4, 44.2) | 3.55 (1.73, 5.85) | 0<0.00010* |
| **Hb** (g/dL), median (IQR)  normal range: 13.7-16.8 g/dL (male), 11.6-14.8 g/dL (female) | 10.0 (8.30, 11.5) | 10.1 (8.40, 11.6) | 9.6 (8.05, 11.4) | 0.17 |
| **Alb** (g/dL), median (IQR)  normal range: 4.1-5.1 g/dL | 2.60 (2.10, 3.10) | 2.78 (2.30, 3.30) | 2.20 (1.80, 2.70) | <0.00010* |
| **Cr** (mg/dL), median (IQR)  normal range: 0.65-1.07 g/dL (male), 0.46-0.79 g/dL (female) | 0.87 (0.65, 1.42) | 0.730 (0.600, 0.955) | 1.66 (1.14, 2.49) | <0.00010* |
| **eGFR** (mL/min/1.73m^2^), n (% of those assessed) |  |  |  | <0.00010* |
| 60+ | 109 (56.4) | 102 (75.0) | 7 (12.3) |  |
| 30–60 | 47 (24.4) | 27 (19.9) | 20 (35.1) |  |
| 15–30 | 15 (7.77) | 1 (0.735) | 14 (24.6) |  |
| 0–15 or requires hemodialysis | 22 (11.4) | 6 (4.41) | 16 (28.1) |  |
| **ALP** (IU/mL), median (IQR)  normal range: 106-322 U/L | 302 (228, 468) | 269 (208, 333) | 498 (353, 847) | <0.00010* |
| **IgA** (mg/dL), median (IQR)  normal range: 93-393 mg/dL | 414 (225, 639) | 540 (382, 711) | 191 (151, 246) | <0.00010* |
| **IgM** (mg/dL), median (IQR)  normal range: 33-187 mg/dL (male), 50-269 mg/dL (female) | 157 (87.6, 262) | 217 (137, 312) | 79 (54, 105) | <0.00010* |
| **IgG** (mg/dL), median (IQR)  normal range: 861-1747 mg/dL | 3597 (1677, 5020) | 4459 (3307, 5632) | 1299 (918.0, 1631) | <0.00010* |
| **IgG4** (mg/dL), median (IQR)  normal range: 11-121 mg/dL | 195 (50.2, 547) | 338 (176, 795) | 32.5 (17.2, 74.8) | <0.00010* |
| **IgE** (IU/mL), median (IQR)  normal range: 0-358 IU/mL | 479 (102, 1890) | 1137 (308, 3270) | 70.3 (37.4, 249) | <0.00010* |
| **CHAP score**, median (IQR) | 6.00 (4.00, 9.00) | 5.00 (3.00, 7.00) | 9.00 (7.00, 11.0) | <0.00010* |

Data are presented as median (interquartile range) or percentages, with statistical significance evaluated using the appropriate tests.* P-values were calculated using the Mann-Whitney U test for continuous variables and the chi-square test for categorical variables. TAFRO: Thrombocytopenia, Anasarca, Fever, Reticulin Fibrosis, and Organomegaly, IQR: Interquartile Range, CRP: C-Reactive Protein, PLT: Platelet Count, Alb: Albumin, Cr: Creatinine, eGFR: Estimated Glomerular Filtration Rate, ALP: Alkaline Phosphatase, IgA: Immunoglobulin A, IgG: Immunoglobulin G, IgM: Immunoglobulin M, ILD: Interstitial Lung Disease. P-values were calculated to evaluate statistical significance between groups. Continuous variables were compared using the Kruskal-Wallis test, and categorical variables were analyzed using the Pearson's chi-square test. A P-value of <0.05 was considered statistically significant.
